# A comparative assessment of cold chain management using the outbreak of circulating vaccine-derived polio virus type 2 as a surrogate marker in Oyo State, Nigeria-2019

**DOI:** 10.11604/pamj.2020.37.313.26152

**Published:** 2020-12-03

**Authors:** Olaniyan Akintunde Babatunde, Muideen Babatunde Olatunji, Olugbade Robertson Omotajo, Olukorede Ifedolapo Ikwunne, Zainab Hamzat, Sunday Thomas Sola

**Affiliations:** 1 Oyo State Primary Health Care Board, State Secretariat, Agodi, Ibadan, Oyo State, Nigeria,; 2 Oriire Local Government Health Authority, Ikoyi-Ile, Oyo State, Nigeria,; 3 Nigeria Field Epidemiology and Laboratory Programme, Asokoro, Abuja, Nigeria

**Keywords:** Immunization, cold chain, management, vaccine, Nigeria

## Abstract

**Introduction:**

inspite of the demonstrable evidence of the preventive and protective ability of vaccines to reduce the outbreak of vaccine-preventable diseases, there are still some significant disease outbreaks recorded in our communities. In some settings, these outbreaks have been linked with poor vaccine management. Therefore, this study was conducted to compare the cold chain practices in Oyo State, Nigeria.

**Methods:**

we conducted a cross-sectional survey among health workers in the local government areas of Oyo State between October and November 2019. Using purposive sampling, we recruited all the 84 routine immunization focal persons for the study. A self-administered questionnaire was used to collect data on cold chain management. Data were analyzed using SPSS version 24 and bivariate analysis was done using Chi-square. Statistical significance was set at p < 0.05.

**Results:**

the mean age of the respondents was 46.4 ± 6.7 years. Most prevalent cadre in the rural facilities was health assistants (87.5%) while Community Extension Health Workers (54.8%) were prevalent in the urban (p = 0.002). The proportion of respondents with adequate cold chain equipment was significantly higher in the urban compared with the rural area. The cold boxes were the only adequate cold chain equipment found in the rural health facilities compared with the urban (p = 0.036).

**Conclusion:**

there was a low proportion of qualified health workers and inadequate cold chain equipment in the rural area compared with the urban facilities. Engagement of skilled health workers and supply of the cold chain equipment are recommended.

## Introduction

Worldwide, about two to three million lives are saved yearly through immunization which is admitted as one of the most successful and cost-effective public health interventions in history [1,2]. In Nigeria, 40% of all deaths among under-5 children are due to Vaccine-preventable diseases (VPDs) [3]. This high mortality has been attributed to poor routine immunization, caused by issues on cold chain system and inadequate knowledge among health workers regarding cold chain management [3]. Poor knowledge and attitude of many health workers had made them to concentrate more on vaccination coverage rather than the quality of immunization services, especially cold chain maintenance, which is important for the reduction of VPDs [4].

In some settings in Nigeria, many health facilities offer immunization without adequate knowledge of vaccine management and may cause possible administration of nonpotent vaccines to the populace [5]. When nonpotent vaccines are administered, the chances of VPDs outbreak are high in such communities. The administration of nonpotent vaccines either during transportation or storage is associated with outbreak of VPDs and suboptimal seroconversion rates [6]. Reports have shown that while vaccination coverage seems to be on the increase, the prevalence of VPDs has not reduced [7]. In recent time, Nigeria has witnessed several outbreaks of circulating vaccine-derived poliovirus type 2 (cVDPV2), even in the settings of high immunization coverage [8]. The detection of these cVDPV2 strains underscores the importance of maintaining high levels of polio vaccination coverage to minimize the risk and consequences of any poliovirus circulation. A situation of high polio vaccine coverage, yet the outbreak of cVDPV2 also underscore the significance of maintenance of cold chain system [8]. The cold chain system is a means for storing and transporting vaccines in a potent state from the manufacturer to the person being immunized [4]. To maintain the potency of vaccines from the manufacturer to the end-users, cold chain management has, therefore, become the essential component of an effective immunization programme [7]. It consists of a series of storage and transport links, all designed to keep vaccines within an acceptable temperature range until it reaches the users [9]. It makes use of human, material and financial resources as well as standards at different levels [10].

As it is important, the cold chain also remains a highly vulnerable point for national immunization programs in developing countries with tropical climates [11]. The success of the Expanded Programme on Immunization (EPI) is therefore muscularly hinged on the cold chain status and hence its management should not be taken lightly. On the other hand, primary health care providers must have adequate knowledge to manage the cold chain [12]. To improve management, the World Health Organization (WHO) has created a set of practice guidelines for different service levels [13]. In recent times, Nigeria registers outbreaks of cVDPV2 even in the areas with high vaccination coverage, the latest was the outbreak recorded in Oyo state. A study conducted in Lagos, Nigeria showed that about three-quarters of the vaccines at the local government level were sub-potent with the condition failing to improve over the three-year study period [14]. With this situation, there is a need to assess the quality of vaccines which was seen as one of the major issues of the National Program on Immunization (NPI) in Nigeria. Therefore, we compared the cold chain system of two local government areas using cVDPV2 as a surrogate marker.

## Methods

**Study location**: the study was conducted in two local government areas (LGAs) located in Ogbomoso, Oyo State. Ogbomoso is the second-largest city in Oyo State and located in the South-western part of Nigeria. Surulere and Ogbomoso North LGAs are rural and urban LGAs respectively. The 2019 projected populations of Surulere and Ogbomoso North LGAs were 219,417 and 306,908 respectively using a growth rate of 3.4% and 2006 population figure as the baseline [15]. The populations of under-one and under-five children in Surulere LGA were 8, 777 and 43, 883 respectively while 12,276 and 61,382 were respectively recorded for under-one and under-five children in Ogbomoso North. There are 28 and 14 health facilities in Surulere and Ogbomoso North LGAs respectively, and these facilities had 84 routine immunization focal persons (RIFPs) and the assistant RIFPs in the two LGAs. There are two types of health facilities in the LGAs; primary health centre and primary health clinic. Each LGA has 10 political wards. There are 10 primary health centres (1 per ward) in each of the LGAs, while the distribution of primary health clinics varies. The health facilities were populated with different cadres of the workforce; Primary Health Care Coordinators (these consist of Medical Officers of Health or in their absence, the most senior health workers), Nurses/Midwives, Community Health Officers (CHOs), Community Health Extension Workers (CHEWs), Medical Laboratory Scientists, Medical Record Officers, Pharmacy Technicians, Health Assistants and Attendants. Routine immunization (RI) services are offered once a week in all the health facilities and are being managed by the routine immunization (RI) focal persons. The two locations were chosen because, in October 2019, there was isolation of a new case of cVDPV2 in Gambari community in Surulere LGA which informed the decision of National Primary Health Care Development Agency and other partners to respond with monovalent Oral Polio Vaccine (mOPV) vaccination. Ogbomoso North LGA was chosen for comparison because 3 out of the 10 wards have boundaries with Surulere LGA.

**Study design and population**: the study was a descriptive cross-sectional survey carried out in all the forty-two (42) health facilities in the two LGAs between October and November 2020. The study population included all the routine immunization focal persons (RIFPs) and the assistant RIFPs who were saddled with the responsibilities of vaccine administration and maintenance of cold chain system at the facility levels. All consenting RIFPs and assistant RIFPs were recruited for the study. RIFPs and assistant RIFPs who were not available during the period of data collection were exempted from the study.

**Sample size and sampling technique**: all the 84 routine immunization focal persons and their assistants in the two purposively selected LGAs were recruited and interviewed.

**Research instrument and data collection methods**: data on socio-demographic characteristics, knowledge on cold chain management practices, storage units and equipment, training and capacity building, temperature monitoring, sources of power supply, funding for cold chain maintenance, workplace infrastructure and job satisfaction of RIFPs and assistant RIFPs were collected using a semi-structure, interviewer-administered questionnaire. Availability, adequacy and functionality of cold chain equipment including the logistics of the health facilities were checked using a direct observational method. Two immunization officers from other LGAs were recruited and trained to assist in data collection. They were trained for two days for 2 hours daily by the principal investigator on the technique of questionnaire administration. The instrument was pretested among RIFPs and assistant RIFPs in local government areas different from the ones used for the main study. The pretest helped to assess the relevance of the questions in eliciting responses from the participants. Difficult questions were rephrased in line with study objectives. Supervision and overall data collection process were done by the principal investigator.

**Measurement of main outcome variables**: knowledge of health workers on cold chain management was assessed using ten questions; respondents were scored according to their responses on different aspects of cold chain management. Each correct response was scored one point while each wrong response was scored zero. The total obtainable score was 10, score above or equal to mean score (6) was categorized as good knowledge. This scoring system agrees with a study on knowledge of health professionals on cold chain management in Ethiopia [16]. The state of infrastructure at NPI office was assessed using 7 questions with the response of “yes” as 1 and “no” as 0. The total obtainable score was 7. A score above or equal to mean score (4) was regarded as a good LGA NPI office.

Job satisfaction was assessed using five items on a five-point Likert scale ranging from very dissatisfied (1) to very satisfied (5). Responses were scored 5,4,3,2 and 1 in that order to obtain the satisfaction score. The sums of the scores for individual respondent were calculated, and the mean of all the scores was determined. The mean satisfaction score was 88.8, while the maximum and minimum satisfaction score was 48.0 and 115.0 respectively. The respondents who scored below the mean satisfaction score were categorized as dissatisfied, while those who scored up to or above the mean were categorized as satisfied.

**Data analysis**: the questionnaires were manually sorted out, entered into a computer and the obtained data were analyzed using IBM SPSS version 25. Descriptive analysis of all the variables measured was first done, and the categorical variables were reported as frequencies and proportions/percentages. Associations between the location of health facility and other categorical variables were assessed using Chi-square. Fisher´s exact test was used when there was an expected value was less than 5. The level of statistical significance was set at p < 0.05.

**Ethical considerations**: approval for the study was obtained from the Ethical Review Committee, Oyo State Ministry of Health. (No: AD13/479/2001). Permission was obtained from the Directors Primary Health Care Coordinator in the two LGAs involved. Written informed consent was obtained from study participants before they could participate in the study.

## Results

Eighty-four questionnaires were administered, seven five were filled and returned giving a response rate of 89.3%.

**Socio-demographic characteristics of the respondents**: the majority (85.3%) of the respondents were 40 years and above. Among the respondents, the proportion of female was 93.3% while the most practiced religion was Christianity (90.7%). The majority of the respondents were married (92.0%) while 86.7% had tertiary education. More than half (56.0%) of the respondents belonged to CHEWs/CHOs cadre and about one-third were health attendants. The majority (73.3%) of the respondents had spent ten years and above in civil service while 92.0% of them had spent 10 years and above in vaccine administration. A higher proportion (82.7%) of respondents received N100, 000 and above as monthly salary. About two-thirds (62.7%) of respondents were in rural health facilities (Table 1).

**Table 1:** socio-demographic characteristics of the respondents of the respondents

Variables	Frequency	Percentage
**Age (years)**		
< 40	11	14.7
≥ 40	64	85.3
**Sex**		
Male	5	6.7
Female	70	93.3
**Religion**		
Christianity	68	90.7
Islam	7	9.3
**Tribe**		
Yoruba	73	97.3
Others	2	2.7
**Marital status**		
Single	6	8.0
Married	69	92.0
**Education**		
Secondary	10	13.3
Tertiary	65	86.7
**Staff cadre**		
Nurse/Midwife	9	12.0
CHEW/CHO	42	56.0
Health assistant	24	32.0
**Grade level**		
< 10	36	48.0
≥ 10	39	52.0
**Years in service**		
< 10 years	20	26.7
≥ 10 years	55	73.3
**Years of vaccine administration**		
< 10 years	6	8.0
≥ 10 years	69	92.0
**Monthly income**		
< 100,000	13	17.3
≥ 100,000	62	82.7
**Health facility location**		
Urban	28	37.3
Rural	47	62.7

**Figure 1:**
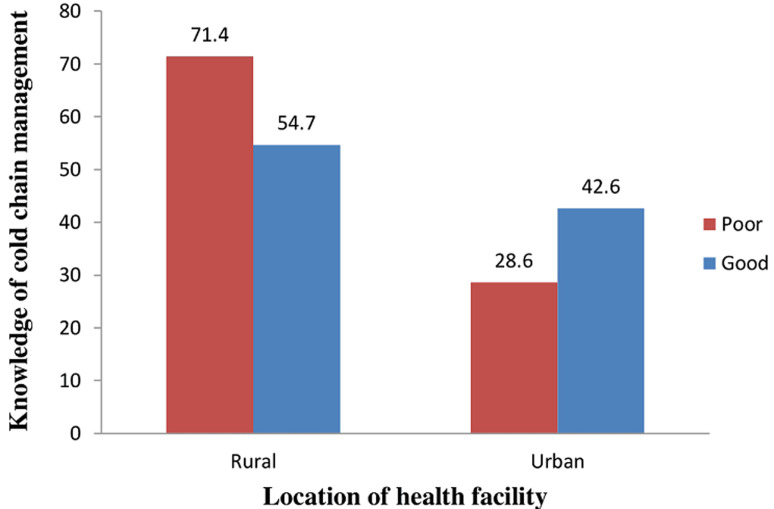
association between knowledge of cold chain management and the location of the health facility of the respondents

**Association between knowledge of cold chain management and location of the health facility of the respondents**: the majority (71.4%) of the respondents in the rural health facilities had poor knowledge while higher proportion (42.6%) of the respondents in the urban health facilities had good knowledge of cold chain management. However, the difference was not statistically significant (p = 0.226) (Figure 1).

**Association between Socio-demographic characteristics and location of the health facility of the respondents**: health assistant appeared to be the highest proportion (87.5%) of cadre staff responsible for vaccine administration in the rural health facilities while CHEWs/CHOs cadre staff had the highest proportion (54.8%) in the urban health facilities (p = 0.002). More than two-thirds (75.0%) of the respondents in rural health facilities were in the grade level of less than 10 while higher proportion (48.7%) of respondents in the urban health facilities were in the grade level of 10 and above. The difference was statistically significant (p = 0.034) (Table 2).

**Table 2:** association between socio-demographic characteristics and the location of the health facility of the respondents

Variables	Location of health facility		P value
	Rural	Urban	
**Age (years)**			
< 40	5 (45.5)	6 (54.5)	0.201
≥ 40	42 (65.6)	22 (34.4)	
**Sex**			
Male	4 (80.0)	1 (20.0)	0.407
Female	43 (61.4))	27 (38.6)	
**Religion**			
Christianity	45 (66.2)	23 (33.8)	0.054
Islam	2 (28.6)	5 (71.4)	
**Marital status**			
Single	5 (83.1)	1 (16.7)	0.275
Married	42 (60.9)	27 (39.1)	
**Tribe**			
Yoruba	45 (61.6)	28 (38.4)	0.269
Others	2 (100.0)	0 (0.0)	
**Education**			
Secondary	7 (70.0)	3 (30.0)	0.607
Tertiary	40 (61.5)	25 (38.5)	
**Staff cadre**			
Nurse/Midwife	7 (77.8)	2 (22.2)	^**^0.002
CHEW/CHO	19 (45.2)	23 (54.8)	
Health assistant	21 (87.5)	3 (12.5)	
**Grade level**			
< 10	27 (75.0)	9 (25.0)	^*^0.034
≥ 10	20 (51.3)	19 (48.7)	
**No of years in service**			
<10 years	14 (70.0)	6 (30.0)	0.428
≥ 10 years	33 (60.0)	22 (40.0)	
**No of years of vaccine administration**			
< 10 years	3 (50.0)	3 (50.0)	0.504
≥ 10 years	44 (63.8)	25 (36.2)	
**Monthly income**			
< 100, 000	7 (58.3)	6 (46.2)	0.470
≥ 100, 000	40 (64.5)	22 (35.5)	

* Significant ^**^ Fisher Exact Test

**Association between cold chain equipment, temperature monitoring and location of the health facility of the respondents**: concerning adequacy of giostyles, a higher proportion (74.2%) of respondents in rural health facilities had inadequate giostyles while a higher proportion (92.3%) of respondents in urban health facilities had adequate giostyles (p < 0.001). A higher proportion (90.9%) of respondents in rural health facilities had a cold box while higher proportion (42.2%) had no cold box in the urban health facilities (p = 0.036). The proportion of adequate frozen icepacks was higher (81.0%) among the respondents in the rural health facilities while greater percentage (44.4%) had inadequate frozen icepacks in the urban health facilities (p = 0.041). A higher proportion (100.0%) of respondents in the rural health facilities had refrigerator equipped without thermometer while in the urban health centres, a higher proportion (52.1%) had refrigerator equipped with a thermometer (p < 0.001).

The proportion of respondents with no thermometer inside the refrigerator was 100.0% in rural health facilities while in the urban health facilities, a higher proportion had thermometer inside the refrigerator (p = < 0.001). In the rural health facilities, majority of the respondents (92.3%) monitored temperature chart once daily while in the urban facilities, the majority of the respondents (68.7%) monitored temperature chart twice daily (p < 0.001). All the respondents in rural facilities did not connect their refrigerator with stabilizer while in the urban facilities majority (45.5%) connected their refrigerator to the stabilizer (p = 0.036). The majority (94.4%) of respondents in the rural facilities kept non-medicinal materials in the refrigerator while the majority (53.5%) in the urban facilities did not keep (p = 0.002) (Table 3).

**Table 3:** association between cold chain equipment, temperature monitoring and the location of the health facility of the respondents

Variables	Location of health facility		P value
	Rural	Urban	
**Functioning vaccine refrigerator**			
No	43(62.3)	26(37.7)	0.833
Yes	4(66.7)	2(33.3)	
**Functioning deep freezer**			
No	46(63.9)	26(36.1)	0.898
Yes	1(60.0)	2(40.0)	
**Adequacy of giostyles for routine immunization**			
Inadequate (< 2)	46(74.2)	16(25.8)	^*^< 0.001
Adequate (≥ 2)	1(7.7)	12(92.3)	
**Cold box**			
No	37(57.8)	27(42.2)	^*^0.036
Yes	10(90.9)	1(9.1)	
**Adequacy of frozen icepacks for the last RI session**			
Inadequate (<8)	30(55.6)	24(44.4)	^*^0.041
Adequate (≥8)	17(81.0)	4(19.0)	
**Refrigerator equipped with thermometer**			
No	20 (100.0)	0(0.0)	^*^<0.001
Yes	23 (47.9)	25(52.1)	
I don´t know	4 (57.1)	3(42.9)	
**Thermometer inside the refrigerator**			
No	13 (100.0)	0(0.0)	^*^<0.001
Yes	28(53.8)	24(46.2)	
I don´t know	6(60.0)	4(40.0)	
**Temperature monitoring chart**			
No	16(94.1)	1(5.9)	^*^< 0.001
Once daily	13 (100.0)	0 (0.0)	
Twice daily	10(31.3)	22(68.7)	
I don´t know	5(41.7)	7(58.3)	
**Refrigerator connected to automatic voltage stabilizer**			
No	6(100.0)	0(0.0)	^*^0.036
Yes	30(54.5)	25(45.5)	
**Non-medicinal materials in the refrigerator**			
No	20(46.5)	23(53.5)	^*^0.002
Yes	17(94.4)	1(5.6)	
I don´t know	7(50.0)	7(50.0)	

* Fisher Exact Test

**Association between capacity building, source of funding and the location of the health facility**: the respondents from the rural health facilities majorly (84.2%) received training on cold chain management less than six months to the time of survey while the respondents from the urban health facilities received the same training between six and twelve months prior survey (p = 0.048). The majority (85.0%) of rural health facilities received routine immunization (RI) logistics from the development partners while about two-thirds (63.3%) of the respondents from the urban health facilities did not receive. All the respondents from the rural health facilities received LGA counterpart fund to support RI while 61.5% of the respondents from the urban health facilities did not receive (Table 4).

**Table 4:** association between capacity building, source of funding and the location of the health facility of the respondents

Variables	Location of health facility		P value
	Rural	Urban	
**Training on cold chain maintenance**			
Nil	27 (57.4)	20 (42.6)	^**^0.048
< 6 months	16 (84.2)	3 (15.8)	
6-12 years	4 (44.4)	5 (55.6)	
**Supervision by LGA team**			
No	4 (57.1)	3 (42.9)	0.510
Yes	43 (63.2)	25 (36.8)	
**Partnersï¿½ RI logistics**			
No	11 (36.7)	19 (63.3)	^**^<0.001
Yes	34 (85.0)	6 (15.0)	
I don´t know	2 (40.0)	3 (60.0)	
**Counterpart logistics fund from the LGA**			
No	20 (38.5)	32 (61.5)	^**^<0.001
Yes	13 (100.0)	0 (0.0)	
I don´t know	5 (50.0)	5 (50.0)	

* Fishers’ Exact Test

**Figure 2:**
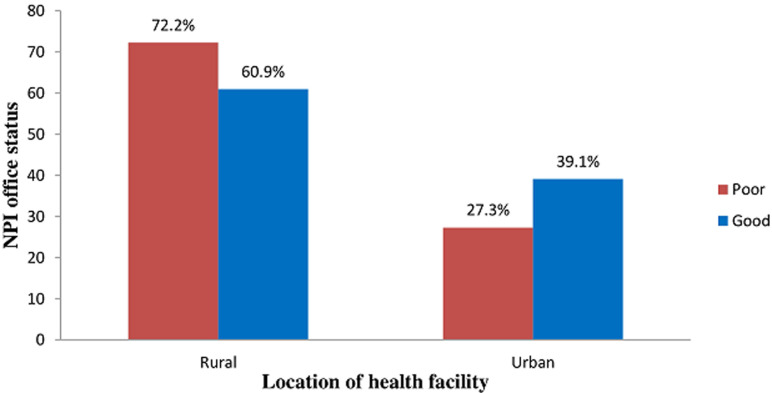
association between the state of LGA NPI office and the location of the health facility of the respondents

**Association between the condition of NPI office status and the location of the health facility of the respondents**: the status of LGA´s NPI office of the majority (72.2%) of the respondents in the rural health facilities was poor while in the urban health facilities, the majority (39.1%) had good LGA´s NPI office. However, the difference was not statistically significant (p = 0.455) (Figure 2).

**Figure 3:**
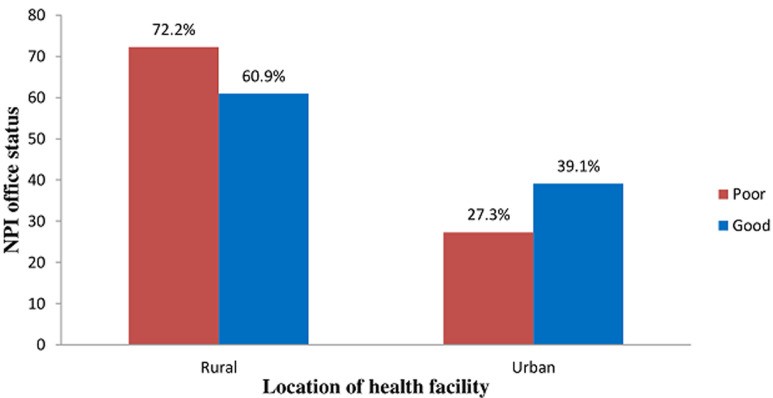
association between power source and the location of the health facility of respondents

**Association between power source and location of the health facility of respondents**: all the respondents (100.0%) in the rural health facilities depended entirely on the public power supply while the majority (51.1%) of the respondents in the urban health facilities depended on public power supply and generator (p = 0.015) (Figure 3).

**Figure 4:**
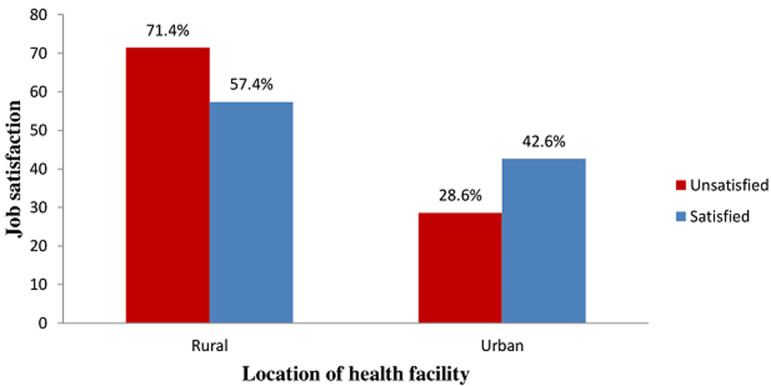
association between job satisfaction and the location of the health facility of the respondents

**Association between job satisfaction and location of the health facility of the respondents**: a higher proportion (71.4%) of the respondents in the rural health facilities was not satisfied with their job while higher proportion (42.6%) of the respondents in the urban health facilities was satisfied with their job. However, the difference was not statistically significant (p = 0.226) (Figure 4).

## Discussion

The study compared the cold chain management practices between a rural LGA where there was an outbreak of cVDPV2 with an urban LGA without an outbreak. The study showed a higher proportion of female involvement in immunization activities, this is anticipated as the majority of the primary health care workers are either nurses or CHEWs and these cadres are largely dominated by women. This gender disparity also corroborated the work of Dairo and Osizimete on vaccine handling and storage in Ibadan, Nigeria [17]. Majority of the respondents have spent more than ten years in the civil service and vaccine administration, this might probably inform the reason while we had about four-fifths of the respondents who were aged 40 years and above. The respondents in this age category represent a set of health workers with the needed experience to do what is needful in terms of giving responses which are considered to be reliable. About four-fifths of the respondents had tertiary education; this is expected as a qualification to become health worker involved the acquisition of post-secondary school certificate. More than half of the respondents were CHEWs/CHOs and this could be inferred from the mode of entry of this cadre, which is not as rigorous as the nursing profession. About four-fifths of the respondents earned N100, 000 (USD 282) monthly. This earning does not reflect the general monthly take home in the civil service of Oyo State but represents health workers´ income with a different salary scale. The respondents in the rural health facilities were about two-thirds of the total sample population which agrees with the pattern of Nigerian population where about 70% resides in the rural areas [18].

Poor knowledge of cold chain management was demonstrated among the respondents in the rural [5] health facilities while in the urban area, [16] higher proportion demonstrated good knowledge. Though not significant, there is a high probability that the health workers in charge of the cold chain system in the rural health facilities lack adequate knowledge of vaccine storage and handling. This could probably account for the recent outbreaks witnessed in the rural LGA. Studies have linked inadequate knowledge of the cold chain management with the outbreak of VPDs [3,5,12]. Therefore, health workers in the rural areas must be prioritized for training on vaccine storage and handling. Our study showed a significant difference in the staff cadre among the respondents in the rural and urban health facilities; with the rural health facilities had highest health assistant cadre while urban is majorly populated with CHEWs/CHOs cadre staff. This is corroborated by a study in Ethiopia where Nurses and Health Officers had better knowledge of cold chain management than diploma holders [5]. With this, there is a tendency of an outbreak to occur in the rural LGA as health attendants are not professionally trained as health workers. With health attendants handling vaccines, wastage rate increases and this could lead to vaccine stock out. In the primary health care organogram, health attendants are at the lowest rung of the ladder with little or no knowledge in vaccine management. Most of these health assistants found themselves as RIFPs and assistant RIFPs because of the corruption in the system and refusal of skilled health workers to work in the rural areas [5] and probably, a reflection of the chronic shortage of manpower experienced in the health sector [5]. Therefore, there is an urgent need for the government to recruit skilled manpower to handle the immunization services, especially in the rural areas.

Concerning grade level, this study documented that about three-quarters of the respondents in the rural health facilities had spent less than 10 years in civil service. The majority of the health workers in the urban facilities had spent 10 years and above. Studies have linked years of experience with performance and knowledge [5,16]. The number of years spent in the service guarantees experience and this may lead to a better understanding of the system [5]. This is invariably found to improve the management of the immunization services, one of the components of the primary health care. The higher proportion of the respondents in the rural health facilities with less than 10 years in civil service could be as a result of rural-urban migration of the experienced health workers while the newly recruited ones are posted to the rural areas [5]. It is therefore incumbent on the government to recruit and provide infrastructure in the rural areas to reduce the rural-urban migration to the barest minimum.

In terms of cold chain equipment, giostyles were inadequate in rural health facilities, compared with urban health facilities with a higher proportion of respondents with adequate vaccine carriers. It could be inferred that when vaccine carriers are not adequate (less than 2), the quality of immunization services cannot be guaranteed. Concerning the national guidelines, one vaccine carrier is meant for immediate service delivery while the second one is to temporarily store vaccines that are not in present use [5]. Any interruption in this process could affect the potency of vaccines. The shortage of vaccine carriers in rural facilities could result from thefts and probably location, as many health facilities are without security. Many studies have attributed the lack of cold chain tools to non-potent vaccines and the potential outbreak of VPDs [6]. It is therefore essential to prioritize rural health facilities for the supply and utilization of vaccine carriers.

The study revealed the presence of cold box and adequate icepacks in rural health facilities while in the urban health facilities, they were in short supply. This aligns with a study conducted in Ibadan, Nigeria [17]. It is understandable that many rural health facilities are located in hard-to-reach areas and scattered settlements without any means of power supply. This is in contrast to what obtains in the urban health facilities where at least a source of power is relatively guaranteed [19]. As a result, cold boxes were mostly used to maintain cold chain in the health facilities, at least for 3 to 4 days. Hence, the presence of more cold boxes in the rural areas. In terms of temperature monitoring, the majority of the refrigerators in the rural health facilities were not equipped with thermometers and voltage stabilizers. Monitoring of temperature charts was mostly done once a day [17]. On the contrary, the urban facilities refrigerators were majorly equipped with thermometer and stabilizers while temperature monitoring chart was done two times a day [19]. Majority of the respondents in the rural facilities kept non-medicinal materials in the refrigerators while in the urban facilities, the majority did not. Failure to monitor the refrigerator´s temperature will not guarantee the vaccine´s potency [20,21]. When non-medicinal materials are kept where vaccines are stored, there is a high tendency for the incessant opening of the refrigerators and this practice could lead to breaking of cold chain system. When the cold chain is broken down, the outbreak of VPDs as a result of administration nonpotent vaccines is inevitable.

The study also revealed a significant difference in the area of capacity building and funding, the majority of the respondents in the rural facilities received training on the cold chain management in less than 6 months prior study while in the urban facilities, pieces of training were majorly received between 6 to 12 months. Studies have shown that well-trained health workers have good knowledge of cold chain management [17,19,22]. High frequency of training in the rural health facilities could be as a result of a high number of health assistants handling immunization services. This cadre of health workers must be frequently trained because of their weak background in immunization. This is consistent with studies in Ethiopia [20] and Malaysia [23]. Majority of the respondents in rural facilities received logistics from the partners and LGAs while it was not so in the urban facilities. Effective cold chain system entirely depends on the stable power supply; unfortunately, the majority of the rural health facilities depend on the public power supply. On the other hand, urban facilities mostly depend on public power supply and generator. It is not surprising that the majority of the outbreaks witnessed in Nigeria and some other developing countries occurred in the rural areas [24,25]. Dependence on public power is associated with non-potent vaccines due to epileptic power supply [25]. When non-potent vaccines are introduced into the communities, even in the areas of high coverage, the epidemic of VPDs can still occur [26].

## Conclusion

The importance of cold chain management is to ensure that potent vaccines are delivered to the final recipients. Without skilled health workers and adequate cold chain equipment, all efforts to maintain good cold chain system remain futile, as clients end up receiving sub-potent vaccines. This is demonstrated when a vaccinated individual come down with VPDs that the specific vaccines were meant to prevent. Addressing the issues of skilled personnel coupled with the identified gaps in the areas of cold chain equipment could be seen as the first step in reducing the incidence of VPDs in the rural communities. **Study limitation**: in this study, data were captured using self-administered questionnaire and on this basis, there could be concealment of some attributes. In addition, the information supplied might not be entirely objective. However, the importance and benefits of giving correct information was explained to them in order to reduce the effects of not supplying correct information.

### What is known about this topic

- Immunization reduces the outbreak of vaccine preventable diseases;
- Vaccine preventable diseases are known to cause increased morbidity and mortality;
- Vaccines are sensitive to temperature changes.

### What this study adds

- Skilled health workers are important in the maintenance of cold chain in rural area;
- Adequate cold chain equipment is vital for the maintenance of cold chain in rural area;
- Health workers’ job satisfaction is crucial in the maintenance of cold chain.
